# Using Bio-Functionalized Magnetic Nanoparticles and Dynamic Nuclear Magnetic Resonance to Characterize the Time-Dependent Spin-Spin Relaxation Time for Sensitive Bio-Detection

**DOI:** 10.3390/s141121409

**Published:** 2014-11-12

**Authors:** Shu-Hsien Liao, Kuen-Lin Chen, Chun-Min Wang, Jen-Jie Chieh, Herng-Er Horng, Li-Min Wang, C. H. Wu, Hong-Chang Yang

**Affiliations:** 1 Institute of Electro-Optical Science and Technology, National Taiwan Normal University, Taipei 116, Taiwan; E-Mails: u20522@hotmail.com (C.-M.W.); jjchieh@ntnu.edu.tw (J.-J.C.); phyfv001@ntnu.edu.tw (H.-E.H.); 2 Department of Electro-Optical Engineering, Kun Shan University, Tainan 710, Taiwan; E-Mails: d92222016@gmail.com (K.-L.C.); hcyang@phys.ntu.edu.tw (H.-C.Y.); 3 Department of Physics, National Chung Hsing University, Taichung 402, Taiwan; E-Mail: chwu@phys.nchu.edu.tw; 4 Graduate Institute of Applied Physics and Department of Physics, National Taiwan University, Taipei 106, Taiwan

**Keywords:** NMR, spin-spin relaxation time, CRP

## Abstract

In this work, we report the use of bio-functionalized magnetic nanoparticles (BMNs) and dynamic magnetic resonance (DMR) to characterize the time-dependent spin-spin relaxation time for sensitive bio-detection. The biomarkers are the human C-reactive protein (CRP) while the BMNs are the anti-CRP bound onto dextran-coated Fe_3_O_4_ particles labeled as Fe_3_O_4_-antiCRP. It was found the time-dependent spin-spin relaxation time, T_2_, of protons decreases as time evolves. Additionally, the ΔT_2_ of of protons in BMNs increases as the concentration of CRP increases. We attribute these to the formation of the magnetic clusters that deteriorate the field homogeneity of nearby protons. A sensitivity better than 0.1 μg/mL for assaying CRP is achieved, which is much higher than that required by the clinical criteria (0.5 mg/dL). The present MR-detection platform shows promise for further use in detecting tumors, viruses, and proteins.

## 1. Introduction

A number of diagnostic platforms have been developed to measure the abundance of biomolecules with high sensitivity. Those platforms enable us to diagnose diseases at early stages and gain valuable insights into their biology at the detection level of the respective systems [1–3]. Some examples include SQUID-based magnetic detection [4–8], magnetic resonance (MR) [9,10], nanowires [11,12], nanoparticles [13], surface plasmon resonance [14], mass spectrometry [15]. Among these, assaying bio-molecules based on magnetic detection has received considerable attention. Magnetic detection can be implemented via the measurements of magnetic relaxation [4,16], remanent magnetization [5], alternating current (ac) susceptibility immunomagnetic reduction (IMR) assay [17], saturation magnetization [18], spin-spin relaxation [19]. The sensors to such a system would include SQUID sensors [20], magnetoresistive sensors [21], micro-MR sensors [22], and magnetic nanotag sensors [23]. However, some of the detection methods are time consuming. It is in our interest to develop a detection platform that is easy to operate and sensitive, enabling us to diagnose diseases at early stages and perform early treatment.

In this work, we report a sensitive diagnostic MR for assaying biomarkers (CRP in this study). In the detection platform we amplify the dynamic MR signals to study the molecular interaction via the measurements of the time-dependent spin-spin relaxation time, T_2_, of protons during the association of Fe_3_O_4_-antiCRP with CRPs. It was found that T_2_ decreases as the concentration of the CRP increases. Additionally, the ΔT_2_ of protons in BMNs decreases as the concentration of CRP decreases, where ΔT_2_ = |T_2_(*t* = 120 min) − T_2_(*t* = 0)|. We attribute this to the stray fields from the magnetization of magnetic clusters that will deteriorate the field homogeneity seen by protons nearby. A detection sensitivity better than 0.1 μg/mL CRP is achieved via the dynamic measurements of T_2_-relaxation. This MR-detection platform shows promise for further use in a broad number of biomedical applications, such as detecting viruses, and proteins, via relaxation measurements.

## 2. Experiments

The dextran-coated magnetic nano-particles were synthesized by chemical co-precipitation [24]. The XRD pattern of the synthesized nanopartices present the high purity for the synthesized Fe_3_O_4_ magnetic particles without other phase such as Fe(OH)_3_ or Fe_2_O_3_. Then the antigoat C-reactive protein was bound onto the dextran on Fe_3_O_4_ particles. Polyclonal goat anti-CRP (Sigma-Aldrich, St. Louis, MO, USA) was covalently bound onto the dextran coated Fe_3_O_4_ particles labeled as Fe_3_O_4_-antiCRP (MagQu Co, New Taipei City, Taiwan) [18]. The BMNs, consisted of anti-C-reactive protein, were dispersed in phosphate buffered saline solution with a pH value of 7.4. Measured with a vibration sample magnetometer, the saturated magnetization of magnetic reagents was 0.3 emu/g measured by vibration sample magnetometer (Model 4500, EG&G, San Francisco, CA, USA), which corresponds to a concentration of 8.5 mg-Fe/mL. A laser scattering analysis (LSA) system (Nanotrac-150, Microtrac, Montgomeryville, PA, USA) was used to obtain the dispersion and average hydrodynamic diameter of biofunctionalized anti-CRP magnetic particles. The hydrodynamic diameter of biofunctionalized anti-CRP magnetic particles is 46.1 nm with a standard deviation of 9.3 nm. The details of the the preparation process and the characterization of BMNs are addressed in references [18,24]. The biotargets of biofunctionalized anti-CRP magnetic particles are human C-reactive protein (CRP).

**Figure 1.**
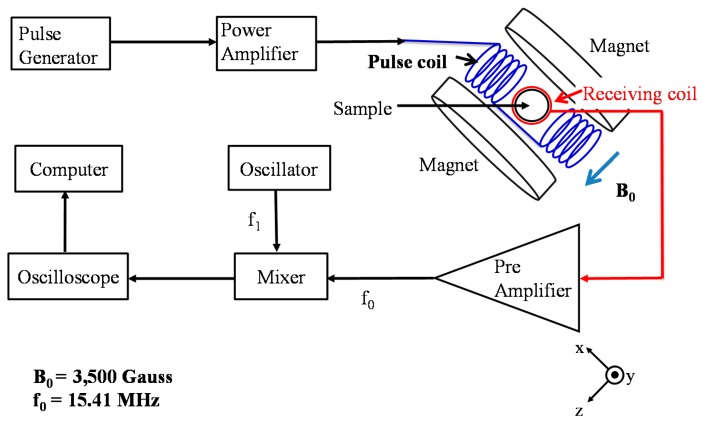
The schematic of the MR detection.

The schematic of the MR detection is shown in Figure 1. The permanent magnet supplies a magnetic field strength of 0.35 Tesla, which corresponds to a resonant frequency of protons at 15.41 MHz. In the spin-echo MR signal detection, a 90° pulse was first applied to the sample via the pulse coil and the spin-echo signals were detected via the receiving coil by applying 180°-pulses. Typical time duration was 25 μs for the 90° pulse and 50 μs for the 180° pulse. The time delay between the 90°-pulse and 180°-pulse was 150 μs, and the time delay between the 180°-pulses (TE time) is 300 μs. The spin-echo signal was amplified and detected via a mixer. The T_2_ relaxation time was measured every five minutes for a total time of 120 min to study the dynamic T_2_ of protons as Fe_3_O_4_-antiCRP becomes associated with CRP.

## 3. Results and Discussion

**Figure 2.**
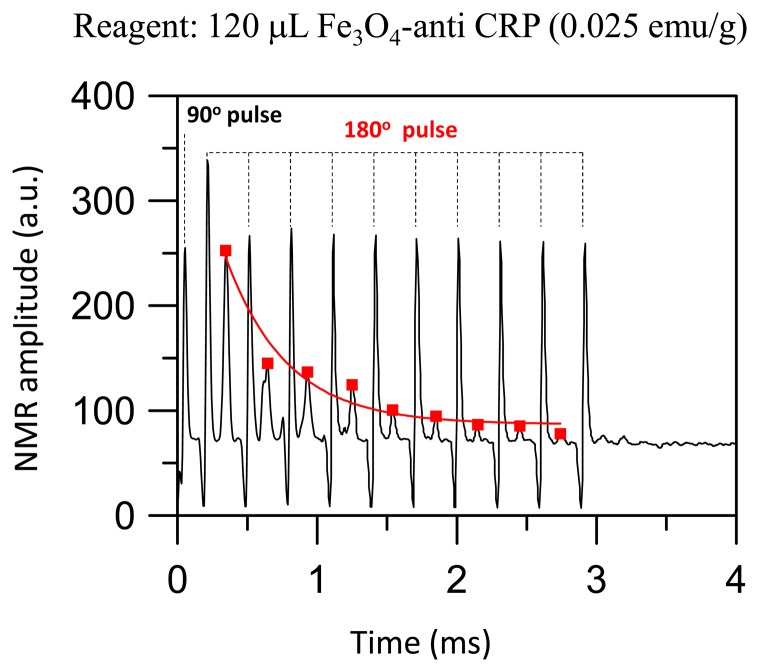
A spin-echo MR signal of protons for a reagent consisted of of Fe_3_O_4_-anti CRP with a saturated magnetization of 0.025 emu/g.

Figure 2 shows a spin-echo MR signal of protons for a 120 μL reagent consisted of Fe_3_O_4_-anti CRP with a saturated magnetization of 0.025 emu/g. The strength of the spin-echo MR signal of protons in BMNs satisfies the relation:

(1) $${\text{M}}_{\text{xy}}(\text{t})={\text{M}}_{\text{xy}}(0)\exp(-\text{t}/{\text{T}}_{2})$$

**Figure 3.**
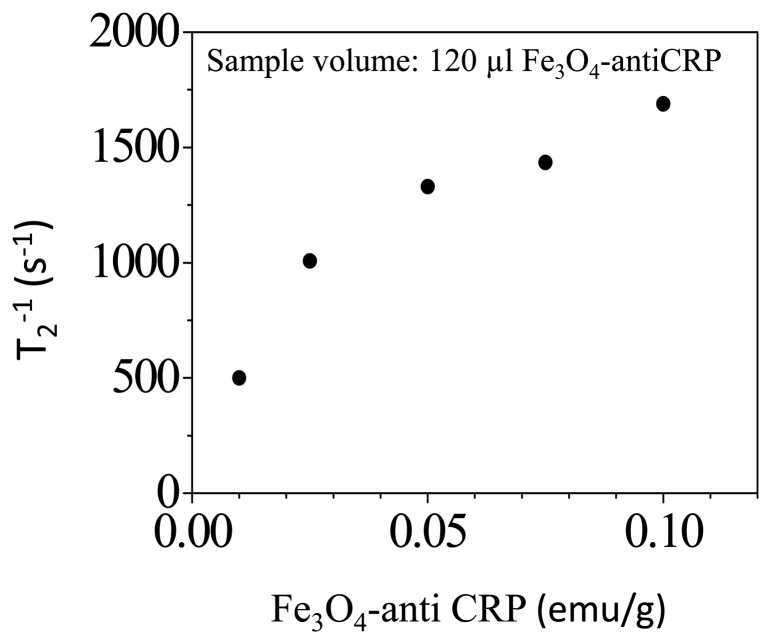
Relaxation rate T_2_^−1^ of protons as a function of Fe_3_O_4_-antiCRP saturated magnetization in unit of emu/g.

The term T_2_ = 992 μs is derived by fitting the data of spin-echo MR signal to Equation (1). Figure 3 shows the relaxation rate T_2_^−1^ of protons in reagents consisted of Fe_3_O_4_-antiCRP as a function of saturated magnetization. In the examination there is no CRP added in to the magnetic reagent. The T_2_^−1^ was 500 s^−1^ as the saturated magnetization of reagents was 0.05 emu/g and the T_2_-relaxation rate increases to 1700 s^−1^ as the saturated magnetization of reagent increases to 0.1 emu/g. Therefore, the T_2_-relaxation rate of protons increases monotonically as its concentration increases. The reagent with magnetic susceptibility of 0.1 emu/g was used to study molecular interactions.

**Figure 4.**
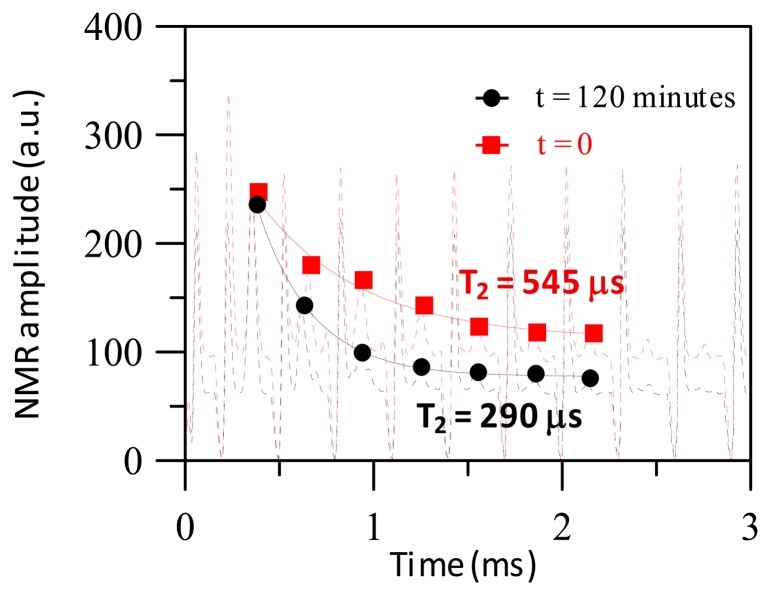
Spin-echo MR signals at *t* = 0 and *t* = 120 min.

Figure 4 shows the spin-echo MR signals of protons when a reagent of Fe_3_O_4_-antiCRP with saturated magnetization = 0.025 emu/g associated with CRP at *t* = 0 before the association and *t* = 120 min after the association. The T_2_ is 545 μs at *t* = 0 and T_2_ decreases to 290 μs after at *t* = 120 min. The decrease of T_2_ at *t* = 120 min in comparison to *t* = 0 is due to the molecular interaction between BMNs with CRP, which causes the conjugation of the magnetic clusters. The stray fields generated from the magnetization of magnetic clusters deteriorate the field homogeneity seen by nearby protons. Therefore the T_2_ relaxation time at *t* = 120 min is shorter than T_2_ at *t* = 0.

**Figure 5.**
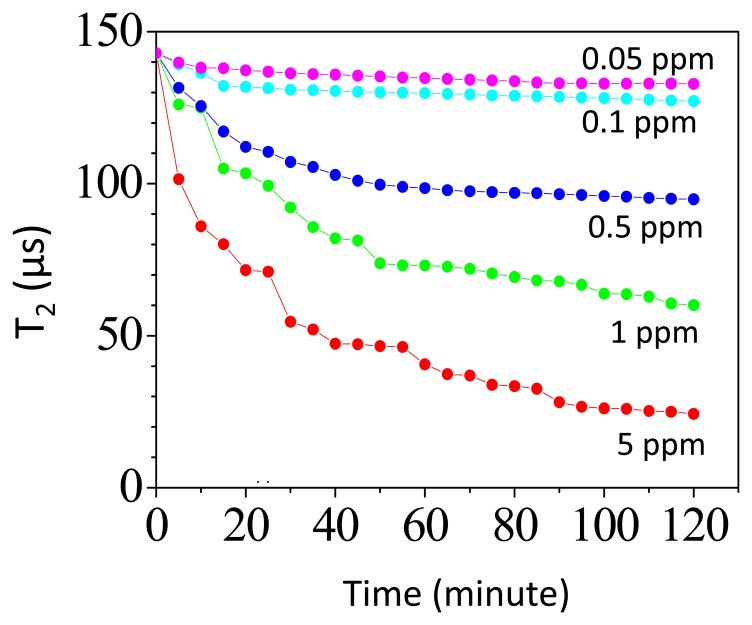
Time-dependent T_2_ of protons in assaying different amount of CRPs.

Figure 5 shows the time-dependent T_2_ in assaying different amount of CRPs. The volume of the reagent is 60 μL and has a saturated magnetization of 0.1 emu/g. In assaying 0.5 ppm CRP, the T_2_ of protons at *t* = 0 is 142 μs and T_2_ decreases to 100 μs at *t* = 120 min. In assaying 0.05 ppm CRP, the T_2_ of protons at *t* = 0 is 142 μs and T_2_ decreases to 136 μs at *t* = 120 min. As we assay the CRP, we find that there is a reduction in T_2_-relaxation time as time evolves. This is due to the formation of the magnetic clusters during the association of Fe_3_O_4_-antiCRP with CRPs. The magnetization from magnetic clusters will deteriorate the field homogeneity seen by protons nearby. Therefore the T_2_-relaxation time of protons decreases as time evolves [19,25].

**Figure 6.**
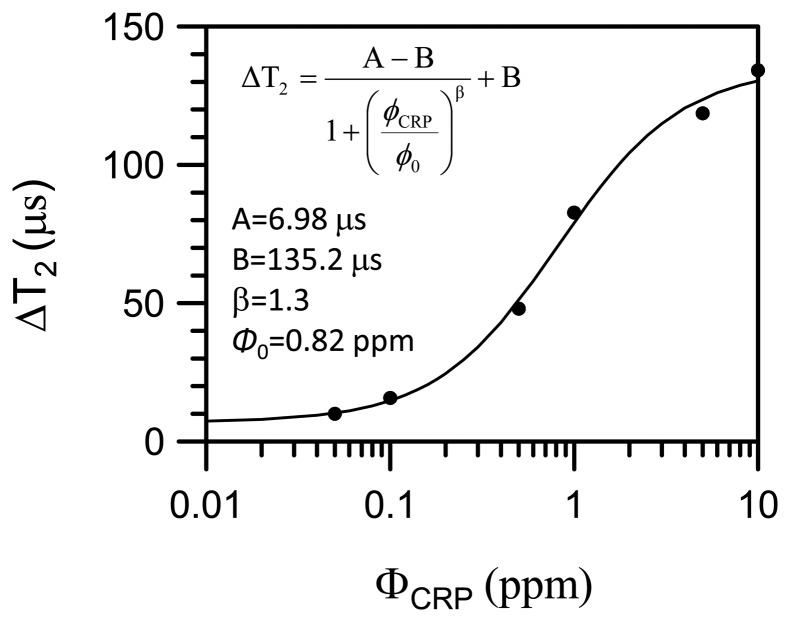
ΔT_2_ of protons as a function of CRP concentrations.

Figure 6 shows the ΔT_2_ of protons as a function of CRP concentrations, where ΔT_2_ = |T_2_(*t* = 120 min) − T_2_(*t* = 0)|. The ΔT_2_ = 135 μs as the concentration of CRP is 10 ppm and the ΔT_2_ decreases to 10 μs as the concentration of the CRP decreases to 0.05 ppm. The ΔT_2_ increases as the concentration of CRP increases. We attribute this to the enhanced molecular interaction that will increase the strength of magnetization in magnetic clusters as the concentration of CRP increases. The enhanced stray fields from the magnetization will increasingly deteriorate the field homogeneity seen by protons nearby. The solid curve is the fitting curve to a logistic function:

(2) $$\Delta {\text{T}}_{2}=(\text{A}-\text{B})/\{1+{\left[\left(\Phi_{\text{CRP}})/(\Phi_{0}\right)\right]}^{\alpha }\}+\text{B}$$

where A and B are fitting parameters in units of micro-seconds and Φ_CRP_ is the concentration of CRP in unit of ppm (or μg/mL) and Φ_0_ is a fitting parameter in units of ppm (or μg/mL). The fitting parameters are: A = 6.98 μs, B = 135.2 μs, α = 1.3 and Φ_0_ = 0.82 ppm. The fitting curve provides a foundation for assaying unknown amount of CRP.

The diagnostic applications using magnetic nanoparticles and microMR were reported [19,22]. That paper addressed the use of MNPs for *in vitro* detection of cellular biomarkers based on the effects of molecular interaction on T_2_-relaxation of protons. That platform showed parallel and rapid measurements from small sample volumes, and a wide range of targets, including whole cells, proteins, DNA/mRNA, metabolites, drugs, viruses and bacteria. The present study reports the time-dependent detection of T_2_-relaxation with the sensing coil wound around the reagent, Fe_3_O_4_-antiCRP. The sensing coil is simple to make and shows high detection sensitivity. The time-dependent T_2_-relaxation of protons is characterized with a reagent of 120 μL within a couple of minutes. To reduce assaying time, we can first mix the reagents with CRPs and let them stand there to complete the association, and then characterize the change of T_2_ using MR, which can be achieved in minutes.

CRP levels in human blood are a key indicator of infectious/noninfectious diseases or acute tissue. The normal concentration in healthy human serum is lower than 10 μg/mL, slightly increasing with aging. Higher levels are found in late pregnant women, mild inflammation and viral infection (10–40 μg/mL), and bacterial infection (40–200 μg/mL) [26]. Examining CRP levels in blood is helpful in the diagnosis of diseases or acute tissue. At present, there are several reported quantitative methods used for assaying CRP, which include enzyme-linked immunosorbent assay [27], radioimmunoassay [28], and immunonephelometry [29], and immunomagnetic reduction assay [17,30]. The present MR detection sensitivity shows high detection sensitivity better than 0.1 μg/mL, which is much higher than that required by the clinical criteria (0.5 mg/dL). The present dynamic MR detection method is robust, easy-to-use and shows potential in the application of detecting a wide variety of biomarkers.

## 4. Conclusions

In this work, we report the time-dependent T_2_-relaxation of protons as the Fe_3_O_4_-AntiCRP is associated with CRP. There is a reduction in the T_2_-relaxation time of protons as time evolves. Additionally, the ΔT_2_ increases as the concentration of CRP increases. We attribute these to the molecular interaction that increases the magnetization due to the presence of magnetic clusters. The local fields created from the magnetization of magnetic clusters deteriorate the field homogeneity seen by protons nearby. A detection sensitivity better than 0.1 μg/mL (0.1 ppm) CRP is verified via the measurements of dynamic T_2_-relaxation. The present MR-detection platform is easy to use and shows promise for detecting tumors, viruses, and proteins.
